# Assessing systemic risk in financial markets using dynamic topic networks

**DOI:** 10.1038/s41598-022-06399-x

**Published:** 2022-02-17

**Authors:** Mike K. P. So, Anson S. W. Mak, Amanda M. Y. Chu

**Affiliations:** 1 Department of Information Systems, Business Statistics and Operations Management, The Hong Kong University of Science and Technology, Hong Kong, China; 2 Faculty of Science, University of Amsterdam, 1098XH Amsterdam, The Netherlands; 3 Department of Social Sciences, The Education University of Hong Kong, Hong Kong, China

**Keywords:** Applied mathematics, Statistical physics, thermodynamics and nonlinear dynamics, Applied physics

## Abstract

Systemic risk in financial markets refers to the breakdown of a financial system due to global events, catastrophes, or extreme incidents, leading to huge financial instability and losses. This study proposes a dynamic topic network (DTN) approach that combines topic modelling and network analysis to assess systemic risk in financial markets. We make use of Latent Dirichlet Allocation (LDA) to semantically analyse news articles, and the extracted topics then serve as input to construct topic similarity networks over time. Our results indicate how connected the topics are so that we can correlate any abnormal behaviours with volatility in the financial markets. With the 2015–2016 stock market selloff and COVID-19 as use cases, our results also suggest that the proposed DTN approach can provide an indication of (a) abnormal movement in the Dow Jones Industrial Average and (b) when the market would gradually begin to recover from such an event. From a practical risk management point of view, this analysis can be carried out on a daily basis when new data come in so that we can make use of the calculated metrics to predict real-time systemic risk in financial markets.

## Introduction

Systemic risk is usually associated with the breakdown of systems due to global events, catastrophes, or extreme incidents. In financial markets, systemic risk can be evidenced by the simultaneous declines in the prices of most or all of the entities in the system due to a large-scale collapse, as opposed to the breakdowns of a few entities^1–3^. As businesses and financial institutions nowadays operate in a complex and dynamic world, their interconnectedness greatly facilitates the propagation of systemic risk. In the 2015–2016 stock market selloff^4,5^ and in the face of COVID-19, countries have shown a significant increase in systemic risk^6,7^. There are various ways to measure systemic risk; among these measures is the Chicago Board Options Exchange’s CBOE Volatility Index, also widely known as VIX^8^, which is designed to capture the level of risk and fear in the market and to generate 30-day-ahead predictions of market volatility^9,10^. Research has previously shown that VIX indicated higher uncertainty and risk aversion in financial markets during several financial crises in the past, such as the Asian financial crisis in 1997 and the collapse of Lehman Brothers in 2008^11^. However, despite the wide application of and research on VIX, some scholars have argued that the index might understate volatility^12^. Given that VIX is derived from the S&P 500 index, it has also been argued that VIX does not cause the realised volatility in the index: in other words, it is not forward looking^13^. Systemic risk has been studied using various approaches, including network analysis^14–19^, spatial-temporal methods^20^, machine-learning methods^21^, and changes in cross-correlations^22^. In this paper, we propose a dynamic topic network (DTN) approach through the combination of two techniques-topic modelling and network analysis-to assess systemic risk in financial markets and also to provide an indication of when the market would slowly recover after the occurrence of a significant event.

The 2015–2016 stock market selloff was partly attributed to the market’s worry on the slowdown of the world economy. The selloff was also partly triggered by the end of the quantitative easing in the United States (U.S.) in 2014, the European sovereign debt crisis and the drop in commodity prices, causing substantial adjustment and turbulence in the stock markets in 2015–2016. Coronavirus disease 2019 (COVID-19) was declared a global pandemic by the World Health Organization (WHO) on 11 March 2020. By the end of October 2021, there had been more than 240 million cases of COVID-19 and 4.8 million deaths caused by the virus, with the numbers expected to keep increasing^23^. The world has been impacted significantly by the pandemic, and the financial markets are among those whose stability has been undermined. A significant number of studies have been conducted, all of which indicate that COVID-19 has brought volatility to different financial markets across the globe^24–27^. The 2015–2016 stock market selloff and COVID-19 are examples of systemic risk, which in finance refers to the risk that a shock or failure event triggers the breakdown of the entire system; the scale could be domestic or transnational. We are interested in applying our DTN method to study the systemic risk during the 2015–2016 stock market selloff and the COVID-19 pandemic.

Research has been conducted to extract insights from textual information: for instance, to explain abnormal volatility in financial markets through news^28^, to forecast market stress and volatility^29,30^, to improve the accuracy of predictions in time series volatility models^31^, and to correlate Internet searches with subsequent movement in stock prices^32^. Sentiment analysis is another way to make use of textual information: for instance, the use of Twitter feeds to predict the Dow Jones Industrial Average (DJIA)^33^ and the use of posts on Weibo to predict Chinese stock price movement^34^. These studies suggested that textual information provides certain predictive powers in financial markets. On the other hand, some studies have used network analysis to depict relations between different entities^35^. Initially, network analysis was applied predominantly in the field of social sciences^36^, but it has now been extended to other fields of study: for example, in the field of medicine to understand the transmission of infectious diseases^37^, the relations between genetics and human diseases^38^, and drug-target interactions^39^ and in the finance field to assess systemic risk and contagion effects in financial markets^40^. Due to the global pandemic, an increasing number of studies have also made use of network analysis to predict and visualise pandemic risk^41–44^ and its influence on financial market connectedness^45–47^.

Although real-time financial news is readily available, research combining textual information and network analysis to predict financial markets is limited. Some similar approaches have previously been proposed. One study, for example, made use of the topic modelling technique to evaluate the temporal change in extracted topics using Japanese news^48^; in that paper, however, articles are grouped by month before the analysis, so the networks might not be responsive enough to detect any sudden changes or potential systemic risk in the market, especially in the case of an unprecedented event such as the global pandemic. Another paper proposed combining the two analytical techniques: In that study, documents were semantically analysed and a similarity network was subsequently constructed^49^; however, the research focused mainly on the visualisation perspective and was therefore not correlated with financial markets for the prediction of market volatility. Given the gap in the existing literature, this paper intends to make two contributions. First, it explores the possibility of quantifying the topological features of extracted topics dynamically. We adopted a rolling window method to obtain updated topological results when new articles are provided; this way we could continuously keep track of and understand how connected the networks were and therefore detect any abnormal patterns immediately. Second, the results might serve as an indicator, on top of other existing financial indices, that topological features could explain systemic risk and opportunity in financial markets in the face of a tremendous change or event.

We used the 2015–2016 stock market selloff and COVID-19 as use cases to demonstrate our DTN approach using all U.S. news articles from Reuters between 1 January 2015 and 31 December 2016, and between 1 January 2019 and 30 September 2020. After data cleaning, we were left with 2,123,284 news articles and 290,771 unique words. The results revealed significantly less connected topic networks in mid May and November 2015, a few months before the downfalls of DJIA in August 2015 and January 2016 during the stock market selloff, and in mid- and late-March 2020, around and after the time when COVID-19 was declared a global pandemic. After exhibiting an abnormal pattern in March and May 2015, network connectedness showed a gradual rebound to normal levels. In March 2020, after exhibiting an abnormal pattern for more than a week, network connectedness reverted to its original level. This might indicate an increase in investors’ confidence in the financial market, which was reflected by the gradual pick up of the DJIA a few months or a few days later. The results also showed the possibility of quantifying and visualising the influence of terms, in particular the emerging term *coronavirus* in mid-March 2020 in our use case, on topic similarity networks in different time periods.

**Figure 1:**
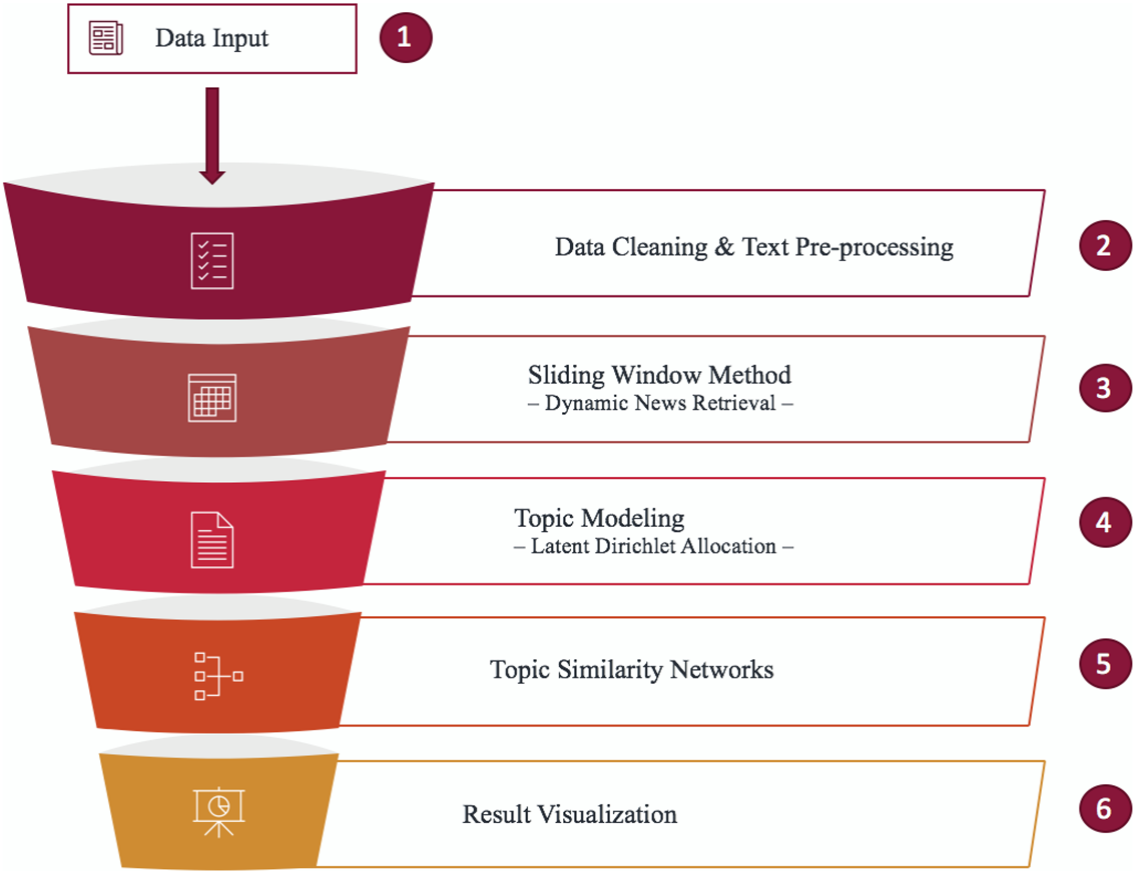
Research design.

Figure 1 shows the design of this research with its six main steps. Step 1 involved collecting news articles of interest for the analysis. In step 2, the input articles were cleaned, lemmatised, and had repeating phrases removed; they were then processed using a sliding window method in step 3. Each article subset was semantically modelled in step 4, and the results were further analysed by constructing topic similarity networks in step 5. Lastly, in step 6, we visualised the final results and compared them to financial indices.

## Results

After the standard text cleaning process, lemmatisation, and the removal of repeated phrases, news articles were sliced by a sliding window of 7 days with a stride of 1 day for dynamic news retrieval to obtain $M_t$ documents at time *t*. Each segment of the $M_t$ documents was semantically analysed using the Latent Dirichlet Allocation (LDA) model, with the number of topics being set to $K=80$^50^. These topics, each represented by words in the corpus and their respective topic-word probability matrices, $\beta _{t}$, served as the input to form DTNs. We used the similarity between $\beta _{it}$ and $\beta _{jt}$ to investigate the topological features of the financial news over time, where $\beta _{it}$ is the *i*-th topic’s word distribution at time *t*. The topological features were summarised using network statistics which can provide insights on financial market evolution, market variation, and systemic risk in financial markets. More detailed description of the construction of the DTNs can be found in the “Methods” section. The results are presented and visualised in the following subsections.

### Visualisation of dynamic topic networks

**Figure 2:**
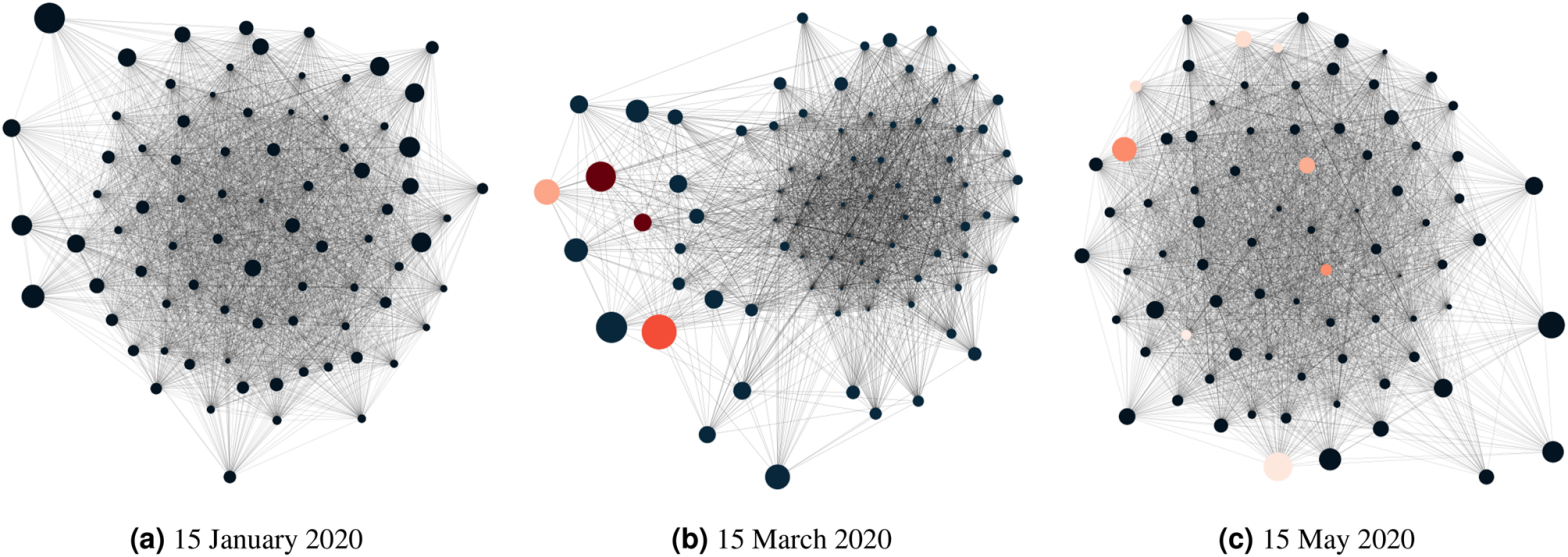
Network graphs at three different dates. Node size represents topic importance, which is proportional to the number the tokens generated by the topic across the whole corpus. For example, a size of 0.2 represents that an estimated 20% of tokens was covered by the chosen topic *i*. If the topic is *coronavirus*-related, its node will be represented by a red gradient, whose colour is mapped to the range between 0 and 0.1 according to the word-topic probability for *coronavirus* in $\beta _{it}$.

To explore the network structures of latent topics from financial news, we present network diagrams where network nodes represent latent topics and edges are formed by the similarity of $\beta _{it}$ among 80 topics. As an illustration, Fig. 2 shows three network structures at different dates, where Fig. 2a–c represent the fifteenth day of January, March, and May 2020, respectively. The first date is before the WHO declared COVID-19 a pandemic on 11 March 2020, and the last two dates are after the announcement. In the figures, each node represents a topic, whose importance is reflected by its node size, proportional to the number of words generated by the topic across the entire corpus or proportional to the topic probabilities in $\theta _t$. If the topic contains the word *coronavirus*, its within-topic frequency percentage is obtained from $\beta _{it}$ and represented by the red gradient according to the percentage.

In Fig. 2a, which shows the topic network on 15 January 2020, it is observed that topics were generally highly connected, without any main deviation of nodes from the main cluster. At this time, the pandemic was not an influential subject, therefore we do not see an influence of COVID-19 on any topics and all nodes are of the same colour. Figure 2b for the topic network on 15 March 2020, on the other hand, shows that the network structure was comparatively less connected and some important topics (with big node sizes) were noticeably farther away from the main cluster. This is not surprising as the nodes with different colours suggest that these topics are *coronavirus* related. With the emergence of this new pandemic-related word and the sudden growth of its influence on news articles, the network structure was altered from dense networks in January 2020 to networks with multiple small clusters in March 2020, causing a sudden drop in network connectedness, as seen in Fig. 2b. Moving forward to 15 May 2020, we observe in Fig. 2c that the term *coronavirus* still existed in various topics, but it no longer showed a significant influence in the news articles, as it did in March 2020, but instead was integrated with other terms. We can also see that the *coronavirus*-related nodes have lighter colours compared to those on 15 March 2020, suggesting a much lower influence of the term within topics as well. The network structure as a whole appears similar to that in January 2020 (Fig. 2a).

### Network connectedness

**Figure 3:**
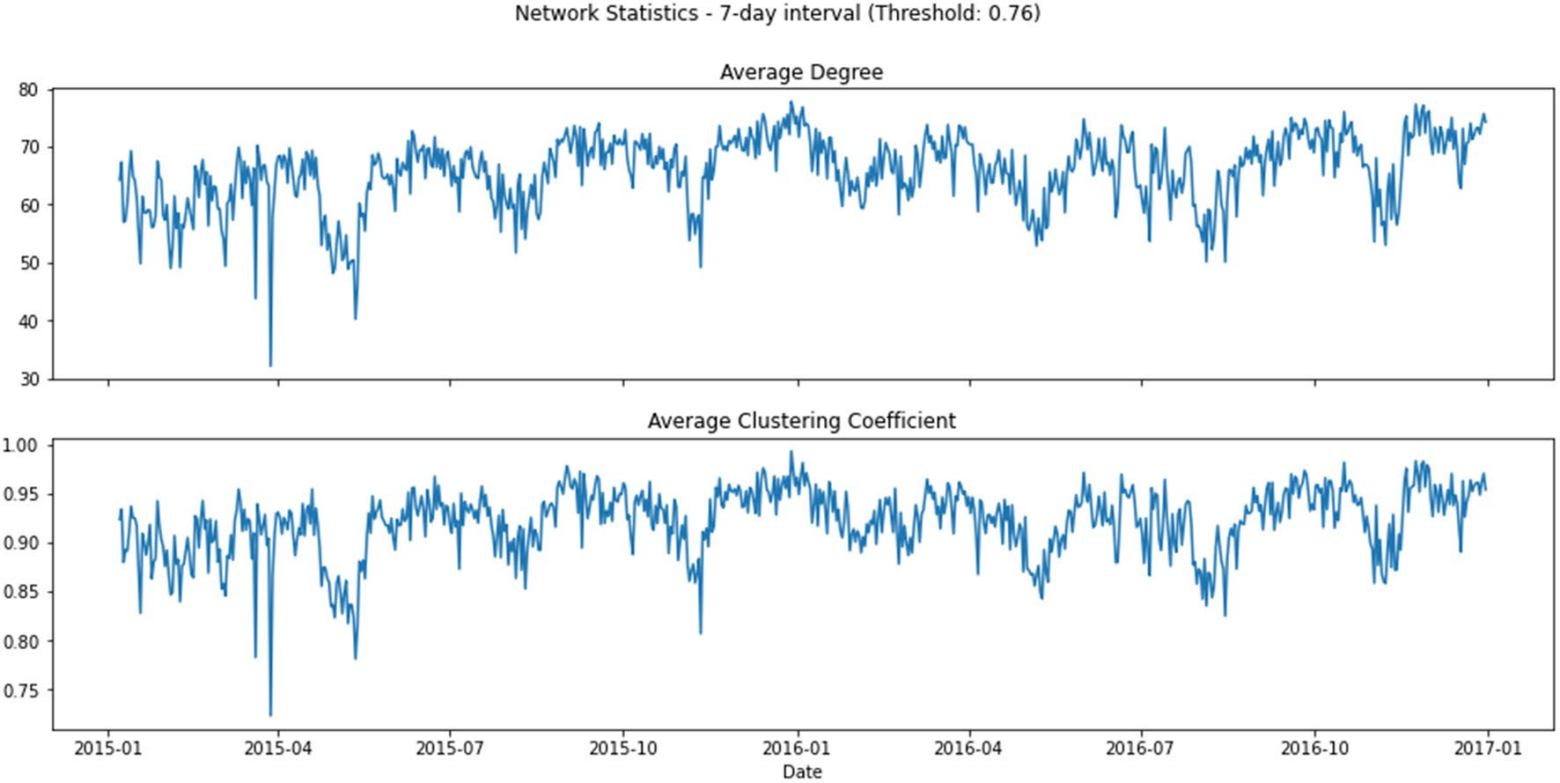
Network statistics, 1 January 2015 to 31 December 2016.

**Figure 4:**
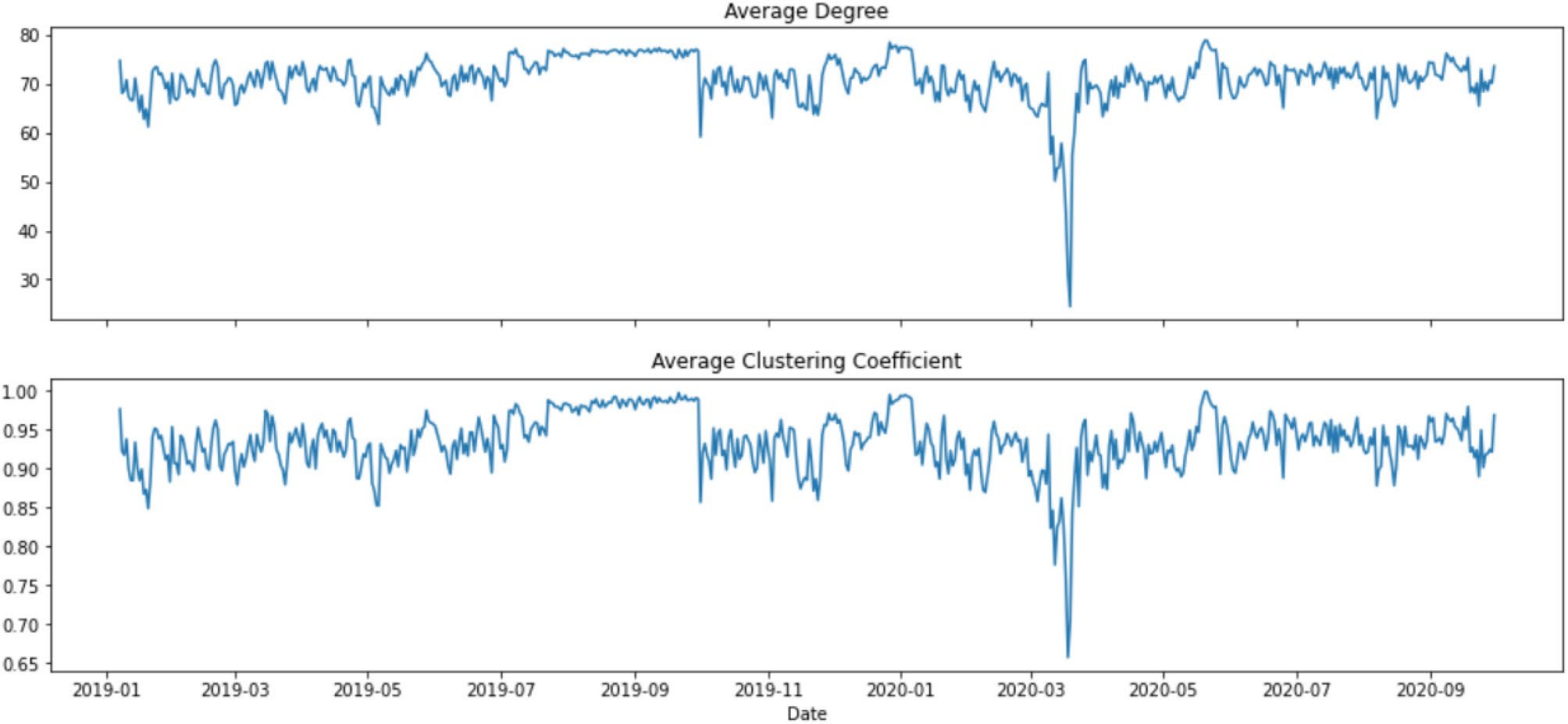
Network statistics, 1 January 2019 to 30 September 2020.

To understand how connected the latent topics were, Figs. 3 and 4 show two network statistics, average degree ($D_t$) and average clustering coefficient ($C_t$), as presented in the “Methods” section, between 1 January 2015 and 31 December 2016, and 1 January 2019 and 30 September 2020. The average degree, $D_t$, in the networks measures how many edges (out of 79, excluding itself) on average link to a node. The higher the average degree, the more dense the network at time *t* will be. Similarly, the average clustering coefficient, $C_t$, measures how big the clusters formed by neighbourhoods of a node of the network are on average at time *t*. In our context, both $D_t$ and $C_t$ measure the level of connectedness of the topic network at time *t*.

In Fig. 3, the connectedness reached the lowest around late March 2015, with the lowest points 20 March ($D_t$ = 43.8, $C_t$ = 0.783) and 28 March ($D_t$ = 32.125, average $C_t$ = 0.723). After around a month of resuming to the normal level, the connectedness began to go downhill again in late April/early May 2015 and reached another local minimum on 12 May ($D_t$ = 40.25, $C_t$ = 0.781). Moving forward, the connectedness reached yet again another local minimum in mid-November, and the drop was in particular obvious for the $C_t$ ($D_t$ = 49.175, $C_t$ = 0.807). There were also some moderately low points in late April/early May, early August and early November in 2016.

As observed in Fig. 4, topics were highly connected (with $D_t$ of around 70) at all time *t* except for March 2020. The two network statistics, $D_t$ and $C_t$, began to show a downward trend on 10 March 2020, and the trend continued until the statistics reached the lowest point on 18 March 2020 for average clustering coefficient, ($C_t$ = 0.657) and on 19 March 2020 for average degree ($D_t$ = 24.475). An interesting phenomenon is that $D_t$ and $C_t$ rebounded quickly to “normal levels” after a sharp drop in mid-March 2020. This structural change in topic network connectedness in March 2020 indicates that semantic features summarised by the DTNs constructed by financial news may give us insights into how financial markets react to unexpected events or even catastrophes. Therefore, studying the relationship between these DTNs and financial market movement may provide systemic risk implications from financial news.

Given the results from the network statistics, we try to understand what the topics were at several time points in the pre-pandemic period, the period when the network statistics started to drop to a low value, and the period when the network statistics returned to a normal level. The fifteenth day of January, March, May, and July 2020 were studied, and the top 30 most salient words are shown in Table 1. A majority of the words are finance related, and some of these words, such as *share*, *target*, and *rating*, are in the list of the most salient words in all four randomly selected dates. One interesting finding was that the word *coronavirus*, which was not the most salient word on other dates, topped the ranking on 15 March 2020, a few days after the WHO declared COVID-19 a global pandemic.

**Table 1:** Top 30 most salient words for the fifteenth day of January, March, May, and July 2020.

Rank	15 Jan 2020	15 Mar 2020	15 May 2020	15 Jul 2020
1	Share	Target	Rating	Target
2	Rating	Share	Price	Rating
3	Target	Market	Share	Price
4	Side	Price	Side	Share
5	Order	Cut	Target	Credit
6	Price	March	Inc	Inc
7	Inc	Nasdaq	Order	Nyse
8	China	Bank	Company	Corp
9	Corp	**Coronavirus**	Corp	Order
10	Year	Rating	Credit	Fitch
11	Bank	Company	Fund	Trump
12	Group	Report	Security	Side
13	Trade	Inc	Bank	Imbalance
14	Stock	World	Source	Company
15	Trump	Service	China	Security
16	Report	Security	Trump	Stock
17	Company	Trump	Service	Group
18	Trial	Oil	Group	China
19	Deal	Event	Year	State
20	Revenue	Credit	State	Bank
21	Agreement	Season	Report	Source
22	Tuesday	Rate	Stock	Holding
23	Credit	Business	Thursday	Year
24	Cut	Thursday	Week	Trade
25	Service	Corp	Market	June
26	State	Week	Cash	Market
27	Energy	Energy	Case	President
28	Market	Year	Rate	Investment
29	Security	April	Revenue	July
30	Sale	China	Usd	Case

The term *coronavirus* is the most salient term only on 15 March 2020.

**Figure 5:**
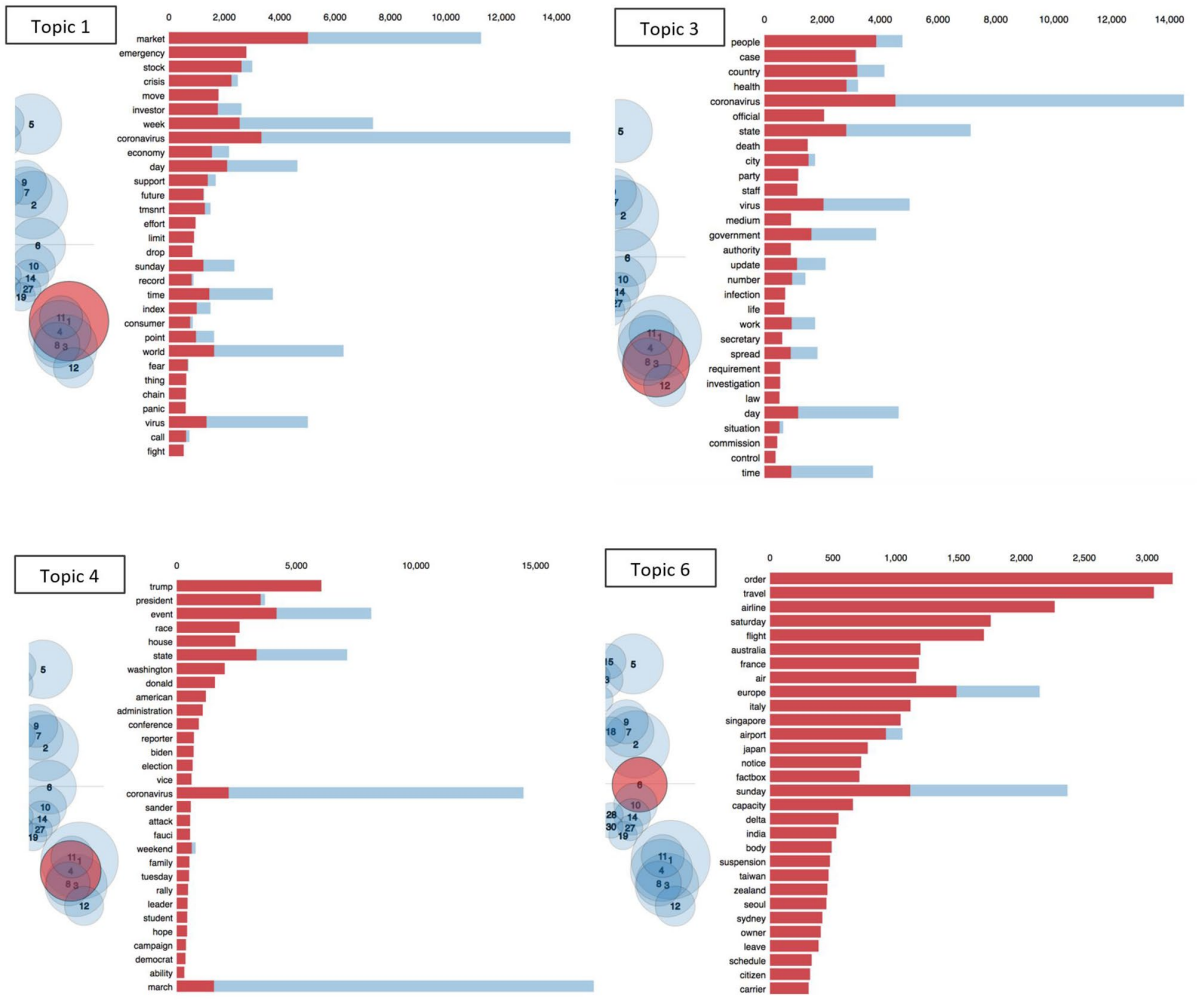
Top 4 most important *coronavirus*-related topics on 15 March 2020.

As mentioned, the word *coronavirus* was one of the most significant words on 15 March 2020, and thus we show the top four most important COVID-19 related topics on that date in Fig. 5 to understand the underlying word distribution for topic *i*, i.e. $\beta _{it}$. The four topics are topics 1, 3, 4, and 6, where the first three topics contain the word *coronavirus* as a major word and topic 6 contains *travel* and *flight* as major words. Topics are represented by bubbles, whose sizes are proportional to their importance and which are clustered together on the basis of their similarities. The four topics, 1, 3, 4, and 6 are highlighted by red bubbles in four diagrams. We can see that *coronavirus* had a significant importance for the selected news period (9–15 March 2020). Also, it comes as no surprise that the bubbles corresponding to the four most important topics are close to each other, indicating a relatively higher across-topic similarity. Topic 1 is related to the financial market in general and how it reacted to the global pandemic. Topic 3 focuses on the societal perspective and the latest development of the virus. Topic 4, on the other hand, links the pandemic to politics in the United States, and Topic 6 shows how coronavirus was affecting the world and the travel industry. The above topic characteristics explain why the connectedness of the topic network can be particularly low when COVID-19, as an emerging global event, tended to have a big influence on financial news.

### Relationships with financial indices

We chose to relate our dynamic topic network statistics to two indices: (1) DJIA: Composed of the 30 largest stocks listed on the U.S. stock exchanges, the DJIA is a good representative of U.S. stock market performance; (2) VIX: This is a derived index from S&P 500; it is widely used by investors and financial institutions to measure market risk and stability and is a good proxy for systemic risk in financial markets.

**Figure 6:**
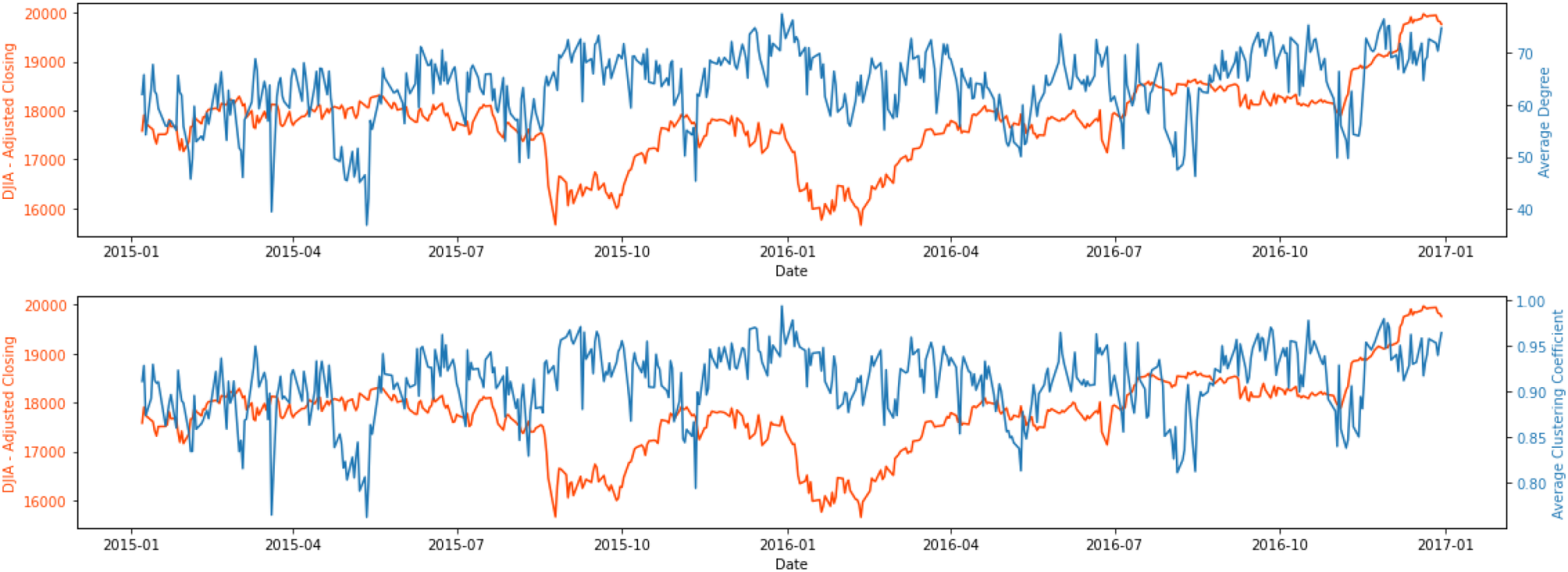
Comparison of network statistics with DJIA, 1 January 2015 to 31 December 2016.

**Figure 7:**
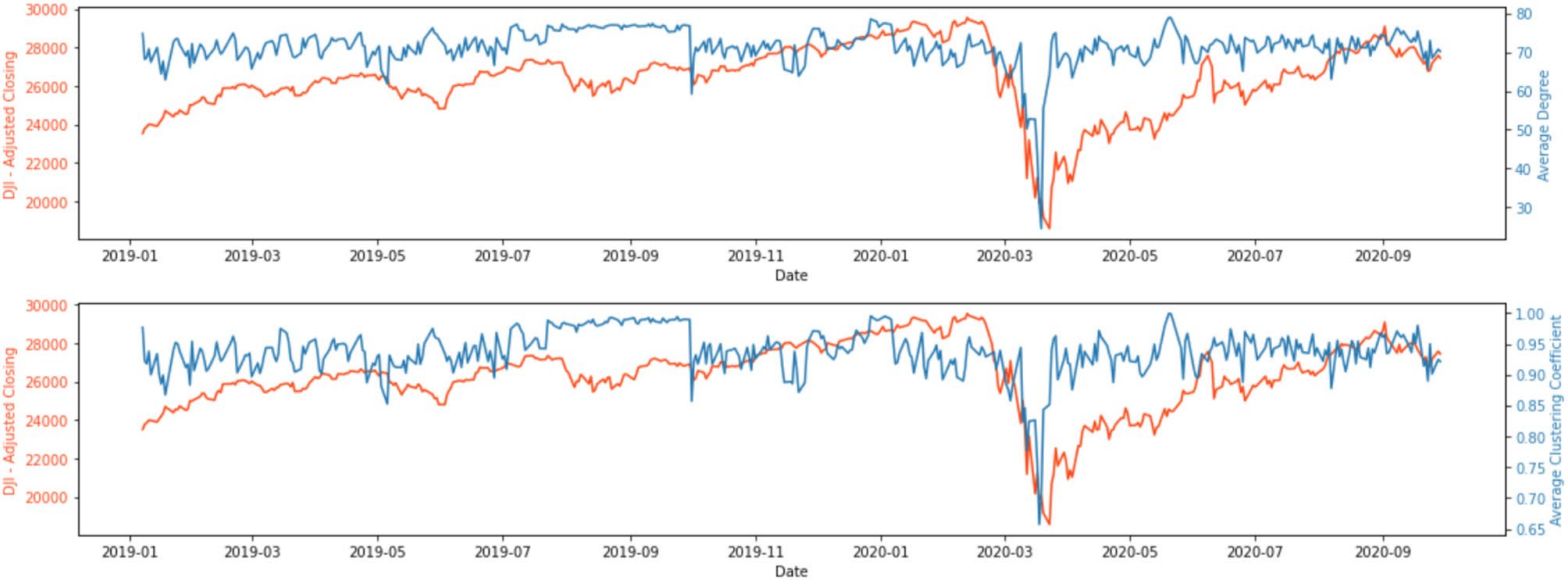
Comparison of network statistics with DJIA, 1 January 2019 to 30 September 2020.

A comparison of the network statistics with the VIX and DJIA gave us some interesting results. Figures 6 and 7 display the network statistics and the DJIA in the same graph. It can be seen that the topic network statistics, $D_t$ and $C_t$, showed two main drops around April/May and November 2015, several months before the drops of DJIA in September 2015 and January 2016, respectively and a general downward trend that aligned with the DJIA in March 2020. During the COVID-19 pandemic, the two network statistics took a sudden dip for a few days and then quickly rebounded to their original levels and became stationary again, whereas the DJIA reached the lowest point of 18,591.93 on 23 March 2020 before gradually recovering to its original level.

**Figure 8:**
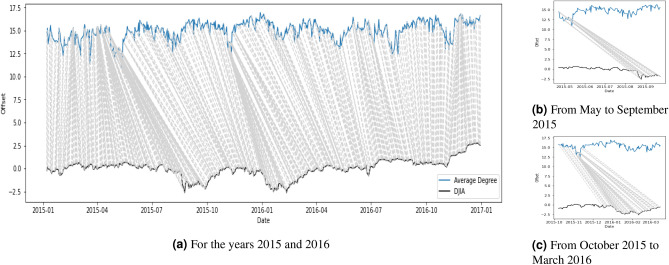
Dynamic time warping: point-by-point comparison between average degree and DJIA for the years 2015 and 2016, both series normalised.

**Figure 9:**
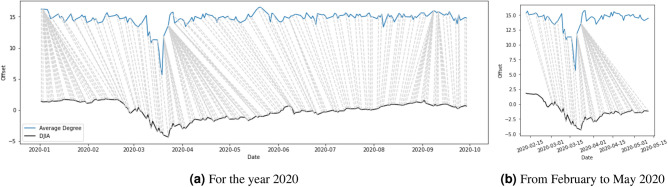
Dynamic time warping: point-by-point comparison between average degree and DJIA for the year 2020, both series normalised.

Given the patterns in Figs. 6 and 7, one might be curious to see if there is any correlation or lead-lag relationship between the time series of the network statistics and the DJIA. Since we were also interested in whether one series was ahead of another, we needed a way to quantify the synchrony between the two series. We adopted the dynamic time warping (DTW) algorithm^51^ to detect the leader-follower interaction. Specifically, we used the DTW algorithm to evaluate the similarity between network statistics and DJIA. It starts with the first time step of the query series and calculates its distance with all data points on the reference series. Then it moves to the second time step of the query series and it repeats the same calculation until the last time step. The best alignment between the two series is found by getting the minimum warping path using the calculations. Figures 8 and 9 show the point-by-point comparison between the average degree, $D_t$, and the DJIA for the years 2015–2016 and 2020 respectively. The grey lines connect the two series on the basis of the shortest Euclidean distance with the implementation of an asymmetric step pattern^51^. In terms of the major events, Fig. 8b,c show that the downturns of average degree in May and November 2015 correspond to the low points of DJIA in early September 2015 and January 2016. Some moderate fluctuations also occurred in 2016, but the similarity relationship was not very obvious. In Fig. 9b, when the average degree dropped to the lowest point on 18 March 2020 and began the pick up to the original level, it provided an indication that the DJIA would also gradually start to pick up in the upcoming months. The direction of the grey lines before 18 March 2020 seems to tell us that the average degree reacted faster than the DJIA. After 18 March 2020, the grey lines showing the same direction as just before 18 March 2020 are even steeper and show stronger evidence that the average degree “led” the DJIA to return to normal levels. The responsive nature of the network statistics potentially provides insights for academics, investors, and financial institutions on when the market would start to recover after an unprecedented event, such as the COVID-19 pandemic investigated in this study.

**Figure 10:**
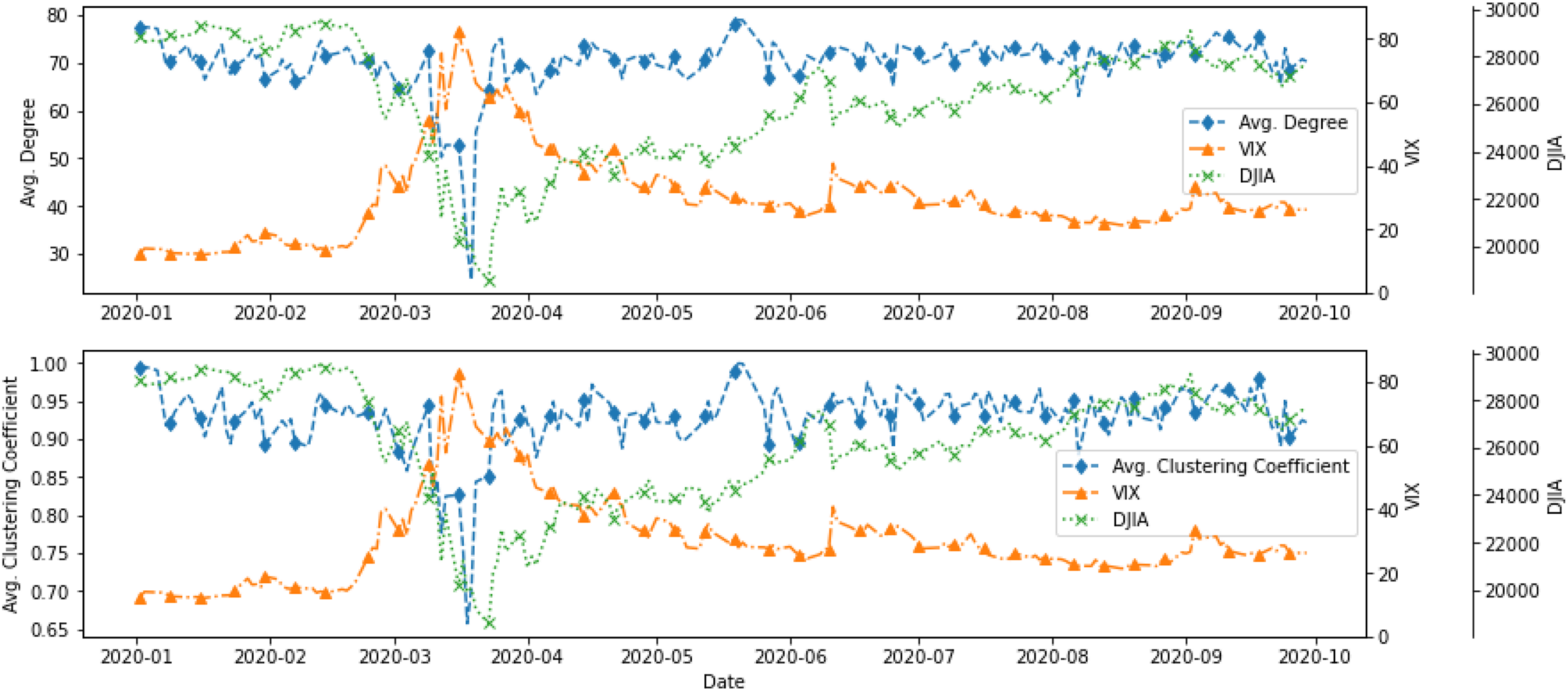
Comparison of network statistics, VIX and DJIA, between 1 January and 30 September 2020.

Figure 10 shows another comparison between the average degree of the topic networks, VIX, and DJIA in 2020. These three series experienced an unusual movement during the pandemic period in February and March 2020. VIX showed an increasing trend starting in late February 2020, while the DJIA exhibited a substantial drop at a similar time until it reached its lowest point of 18591.93 on 23 March 2020. The two topic network statistics, $D_t$ and $C_t$, started to show a drastic decrease in early March, dropping to their lowest value on 18 March 2020 before responsively returning to their original value a few days later.

## Discussion

The severe downturn of financial markets or systemic risk can be attributed to different events or financial incidents. A possible incident is related to credit markets and a substantial increase in default probabilities in a short period, for example, in the financial tsunami in 2008. There is common scientific evidence of having higher systemic risk from a significant increase in financial network connectedness^45,52–54^. The idea of using the topic network connectedness of latent topics to capture possible systemic risk is explained in Fig. 2. When an event that is of great financial relevance is evolving, there can have a transition in the topic network structure from Fig. 2a,b, where we see the emergence of event-related words (in this case pandemic-related words), causing the quick formulation of small clusters of new topics and a sudden drop in the topic network connectedness. The small clusters of new topics may signal unexpected responses in the financial markets to the big events which can cause big impacts to the financial markets. To further elaborate the use of the dynamic topic networks as a possible tool to provide early warning signals for severe financial downturns, we have included the use cases of the 2015–2016 stock market selloff and the COVID-19 pandemic. We find a substantial drop in the topic network connectedness in March and May 2015 before seeing a substantial adjustment in the DJIA in August 2015. We also see a sudden drop in the topic network connectedness in March 2020 during the COVID-19 pandemic.

To understand the underlying topics in news articles and whether they could provide an early assessment of systemic risk in financial markets, we made use of LDA for topic modelling^55^, and the latent topics were used to construct DTNs on the basis of topic similarities^36^. The final outputs (i.e. metrics of network connectedness) were related to benchmark market indices to investigate the predictive power of our approach. Using the 2015–2016 stock market selloff as a case, Fig. 3 reveals a sudden drop in the topic network connectedness in March and May 2015. These significant decline in topic network connectedness can be an indication of the propagation of fear in financial markets which is reflected in the evolution of new influential topics in the corpus. As seen in Fig. 6, following the significant drops in the topic network connectedness in about three months, we see a steep downturn in DJIA in August 2015. Using COVID-19 as a case having worldwide impacts, we conclude that DTNs and their network statistics could be auxiliary measures to detect systemic risk and, in addition, could provide an opportunity to determine when a market might pick up after a tremendous change in the financial markets due to extraordinary events or catastrophes. As seen in Fig. 4, the topic networks began to be less connected on 10 March 2020. As we used a sliding window length of 7 days, this means the analysis on that date included news articles from 4 to 10 March 2020. This might be an indication that investors already foresaw instability in the financial market even before WHO declared COVID-19 a global pandemic on 11 March 2020, and this loss in confidence was captured in the news articles prior to the slump in the DJIA. Slightly more than a week after the global pandemic declaration, the topic network statistics started to pick up again and rapidly returned to their original level. The DJIA exhibited a similar pattern a few days after the recovery of the topic network statistics, the only difference being that the increasing trend was much slower.

As stated in the “Introduction”, VIX is one of the widely recognised indices for measuring market volatility and a main risk indicator for numerous financial institutions and investors to refer to, although some have argued that it might have limited predictive power^12,13^. In this research, we included VIX for comparison with the network analysis results. As can be seen in Fig. 10, VIX started to drop at a similar time to when the network statistics rose. Same as the DJIA, however, the rate was comparatively slow due to the fact that it was highly related to the derivative products on the S&P500 index^8^, and it was not until May 2020 that VIX began to reach a plateau. The topic network statistics are much more responsive measures compared to VIX, possibly because news articles are real-time information that contain a variety of insights and latest events from around the globe. Financial news, in particular, is a useful source that captures and encapsulates investors’ confidence in the market. The increase in the topic network statistics a week after the global pandemic declaration might have provided an indication that investors were experiencing less uncertainty and becoming less anxious about future movement in the financial market, which could therefore subsequently explain the gradual increase in the DJIA that followed a few days later.

The point-by-point comparison in Figs. 8 and 9 also suggest that the average degree, $D_t$, was ahead of the DJIA (i.e. possibly showing a leading time series signal for future movement of the DJIA). During the COVID-19 pandemic, When the average degree began to recover in mid-March 2020, it provided an indication that the DJIA would also slowly pick up in the coming month. This further highlights the possibility of using news articles to ascertain when the recovery of global stock markets might happen after a period of crash and thus detect opportunities in the financial market. The dynamic information in news articles can supplement other indices, such as VIX, in guiding investors and financial institutions to have better predictive power in financial markets, especially on systemic risk management. It is important to note that the DTW algorithm tries to match the patterns by constructing the optimal warping paths and does not necessarily imply causality. Nevertheless, it provides us with a glimpse of the similarity between the two series and how time indices in one series could be matched to those in the other series.

This paper shows the possibility of combining the topic modelling and network analysis of textual information to give predictions in the financial market. A limited number of studies have attempted to combine the two methods to understand news articles but from other perspectives^48,49^. This paper looks at the methodology from the perspective of constructing DTNs and uses topic network statistics to capture network connectedness hidden in text and financial news. We also relate our topic network results to the DJIA and VIX. The results show the potential of using this novel approach to predict the influence of the pandemic on financial risk. The COVID-19 pandemic induced pandemic-related salient words in the news articles on 15 March 2020, and it is evident from Fig. 2 that their presence caused topic similarity networks to be less coherent. The word *coronavirus* was still in the corpus after two months; however, it was much less influential and began to become part of other bigger topics. Moreover, our proposed topic network approach allowed us to easily quantify the influence of latent topics dynamically, as in Fig. 4. Through the combination of topic modelling and network analysis, we have gained a better understanding of the influential words and topics in selected time periods.

This paper takes the COVID-19 pandemic as a use case. The paper^45^ documented the extraordinarily high financial network connectedness due to the impacts of the COVID-19 pandemic. In addition, the papers^46,47^ presented evidence that the pandemic network connectedness can lead the financial network connectedness. The above research documented the possible contagion or transmission of the pandemic risk to financial risk. Possible channels of the transmission can also be found in^24^. The implication of our methodology does not limit to the COVID-19 pandemic. To illustrate further insights of the dynamic topic networks on financial risk evolution, we considered another use case, the 2015–2016 stock market selloff to illustrate how topic network statistics may generate early warning signals of DJIA’s downfalls a few months prior. As far as we know, there is no existing result on studying the relationships between dynamic topic networks derived from financial news and financial or systemic risk. Our paper aims at filling the research gap to showcase the use of the dynamic topic networks as a supplementary tool for providing early warning signals of extraordinary financial market downturns or systemic risk.

Latent topics from financial news reflect contemporary issues or events relevant to the financial markets. Our findings present evidence that new latent topics may emerge suddenly and become influential to financial investors. The emergence of new topics can induce small clusters of new topics as seen in Fig. 2. This paper demonstrates that by extracting the dependence in the latent topics to form dynamic topic networks, we can provide hints to extraordinary financial market downturns or systemic risk. We have presented evidence from the financial market adjustment in 2015 and the COVID-19 pandemic. For applying the dynamic topic network methodology to other financial markets, we are recommended to use corpora relevant to the financial markets of interest. For example, if we want to infer and trace the systemic risk in the financial market in Hong Kong, we can construct the dynamic topic networks using the news corpus of Hong Kong or Asia. From the risk management point of view, the dynamic nature of the research framework means that the analysis can be continuously updated to generate predictions on a daily basis, provided that the latest financial news articles are available. Just as we quantify the influence of the COVID-19 pandemic in this study, in the future, we could also capture and quantify the sentiment of financial institutions and investors through news, observe if any anomalies exists in world events that could change investors’ confidence, and dissect the fluctuations via observation of topic network connectedness over time. We can also attribute these changes in network connectedness to particular words to provide a comprehensive and scientific view of the semantic dynamic of the news. Further research will be conducted to solidify the findings and extend the applicability of the proposed research framework.

## Methods

This section describes the methodology of the research. Each step corresponds to the research design in Fig. 1. Our data analysis is entirely based on Python. We used gensim for most of the natural language processing, in particular the LDA that we heavily focused on in this research. To create network graphs, we used networkx. To create topic distribution graphs in Fig. 5, we used pyLDAvis. For the DTW algorithm, we used dtw-python. All other visualizations are made with the package matplotlib.

### Data

For this research, we collected U.S. news articles between 1 January 2015 and 31 December 2016, and 1 January 2019 and 30 September 2020 from Reuters. Table 2 shows a few examples of financial news with the posting date, headline, and body text.

**Table 2:** Examples of news articles.

Posting date	Headline	Body text
1 Jan 2020	A major shake-up for oil and shipping	Tougher rules on sulphur emissions from ships came into effect on Wednesday, in the biggest shake-up for the oil and shipping industries for decades .
1 Jan 2020	U.S. auto safety agency to probe fatal Tesla crash in Los Angeles	The U.S. National Highway Traffic Safety Administration (NHTSA) said late on Tuesday it will investigate a fatal Dec. 29 Tesla Inc $<\hbox {TSLA.O}>$ crash in Los Angeles that killed two people
1 Jan 2020	Brookfield Ontario 189 MW Prince wind power plant returns to service—Ontario IESO	Brookfield Ontario 189 MW Prince wind power plant returns to service, the Ontario IESO said on Tuesday
1 Jan 2020	Iran’s Khamenei strongly condemns U.S. attacks in Iraq—TV	Iran’s Supreme Leader Ayatollah Ali Khamenei strongly condemned U.S. attacks on Iran-allied militia group in Iraq, Iranian state TV reported on Wednesday .

### Text cleaning

For each article, the headline and the body text were combined to form the full text. Standard text-cleaning procedures were carried out, including the removal of white spaces, non-alphabetical characters, and stop-words and the conversion of all words to lower case. Only nouns and proper nouns (part-of-speech tags: NOUN and PROPN)^56^ were retained before applying lemmatisation. Words that appeared only once in a given period are removed from the constructed dictionary. On the other hand, there were numerous repeated phrases in the articles: for instance, ‘Reporting by ...’, ‘For company coverage: ...’, and ‘Click the following link to watch video: ...’, and so on. These phrases do not add value to the analysis and were therefore removed using regular expressions. The final cleaned version consisted of 2123284 news articles with 290771 unique words, and this served as the input for the topic modelling. Tables 3, 4, 5, and 6 show the number of articles and unique words per month for 2015, 2016, 2019, and 2020, respectively.

**Table 3:** Number of articles and unique words in 2015.

Month	Number of articles	Number of unique words	Month	Number of articles	Number of unique words
2015/01	63,193	49,834	2015/07	68,813	53,002
2015/02	66,933	51,453	2015/08	60,503	49,297
2015/03	73,658	53,916	2015/09	62,556	49,616
2015/04	70,974	53,780	2015/10	71,587	52,612
2015/05	67,986	53,293	2015/11	67,638	52,347
2015/06	68,767	52,812	2015/12	51,148	46,441

**Table 4:** Number of articles and unique words in 2016.

Month	Number of articles	Number of unique words	Month	Number of articles	Number of unique words
2016/01	57,453	40,006	2016/07	61,567	38,709
2016/02	65,954	41,828	2016/08	61,607	40,114
2016/03	62,898	41,377	2016/09	53,616	38,768
2016/04	65,097	40,047	2016/10	55,694	37,642
2016/05	66,997	40,685	2016/11	60,381	39,336
2016/06	64,077	39,685	2016/12	46,552	34,559

**Table 5:** Number of articles and unique words in 2019.

Month	Number of articles	Number of unique words	Month	Number of articles	Number of unique words
2019/01	27,932	24,543	2019/07	27,876	25,024
2019/02	26,776	24,419	2019/08	29,152	24,424
2019/03	29,819	25,294	2019/09	27,810	24,451
2019/04	27,495	24,368	2019/10	31,244	26,356
2019/05	29,827	25,848	2019/11	28,577	24,420
2019/06	27,464	23,795	2019/12	24,305	21,665

**Table 6:** Number of articles and unique words in 2020.

Month	Number of articles	Number of unique words	Month	Number of articles	Number of unique words
2020/01	27,942	24,096	2020/07	27,965	23,412
2020/02	27,307	23,607	2020/08	26,583	22,434
2020/03	43,727	27,872	2020/09	28,536	23,851
2020/04	31,367	23,484			
2020/05	23,707	21,672			
2020/06	32,224	23,359			

### Sliding window

This study adopted a sliding window approach in which news articles in a given window length were extracted, semantically modelled, and analysed with a topic similarity network; then, the window would slide to the next period. These steps were repeated until the window reached the last available date of the provided news articles. In this study, the window length was set as 7 days with a stride of 1 day so that the LDA in the next step could capture the latent topics with enough news articles while also being able to reflect changes in a responsive manner. Specifically, on day *t*, we used the documents on day $t-6$ to day *t* to perform the LDA. By doing so, we intended to observe how the connectedness between latent topics changed over time.

### Latent Dirichlet Allocation

Topic modelling was used to understand the latent topics behind news articles. Among the various methods available, LDA is widely used^55^. This generative probabilistic model represents a document as a random mixture of latent topics, where each topic is further characterised by a distribution of words. Suppose we have *N* words in the corpus and $M_t$ documents on days $t-6$ to *t*. Given $M_t$ documents as input at time *t*, let *K* be the number of latent topics in the documents. The $M_t$ documents contain a sequence of words which are assumed to be generated from a Dirichlet mixture of multinomial distributions under LDA in two steps. For each document at time *t*, the first step is to generate a latent topic probability vector $\theta _t$ from a *K*-dimensional Dirichlet distribution, denoted by Dirichlet$_K(\alpha _t)$ with a $K \times 1$ parameter vector $\alpha _t$. In the second step, given the latent topic probability vector $\theta _t$, we first simulate a latent topic vector $z_n$ for the *n*-th word. Then, for $z_n=i$, where *i* is a topic index between 1 and *K*, we generate the *n*-th word from the multinomial distribution with the $N \times 1$ probability vector $\beta _{it}$. Combining all *K* vectors $\beta _{it}$, $i=1, ..., K$, we can form a $K \times N$ topic-word probability matrix at time *t*. We are interested in estimating $\alpha _t$ and $\beta _t$ using LDA to determine the topological features of the latent topics of financial news.

In an unsupervised learning setting, there is no ground truth of how many topics *K* are behind the articles. Literature has suggested the use of a large number. In previous studies, *K* was arbitrarily set to a large number^50^. In this paper, we selected 80 as the number of topics.

### Topic similarity network

The primary focus of network analysis is to better understand relationships between entities. This study intended to take the output from the LDA model as the input into the topic similarity networks so that we could further understand topic connectedness and how it changes over time.

#### Measuring topic similarity

As previously mentioned, topics are represented as distributions of words and captured in the matrix $\beta$. Therefore, the similarity between two topics can be measured by comparing their word distributions. Kullback-Leibler (KL) divergence is often used when calculating the similarity between distributions. Although it is intuitively used to measure the distance between probability distributions, it is not a true metric given its asymmetry and the fact that it does not satisfy the triangle inequality. Alternatively, this study adopted Jensen-Shannon (JS) divergence, which is based on KL divergence, to calculate a normalised score. In other words, JS divergence can be considered as a symmetric and smoothed version of KL divergence. Equation (1) shows how JS divergence between probability distributions *P* and *Q* is calculated:

1 $$\begin{aligned} JS(P||Q) = \sum \frac{1}{2} P\times log\frac{P}{M(P,Q)} + \frac{1}{2} Q \times log\frac{Q}{M(P,Q)}, \end{aligned}$$

where

2 $$\begin{aligned} M(P,Q) = \frac{1}{2} (P + Q). \end{aligned}$$

When $P=Q$, the JS divergence will be zero. The smaller the *JS*(*P*||*Q*), the more similar *P* and *Q* will be.

#### Topic network

**Figure 11:**
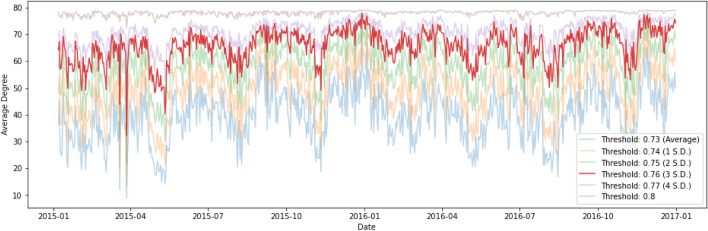
Threshold sensitivity for the period of 2015 to 2016.

A topic similarity network $$G_{t} = (V_{t},E_{t})$$ can be constructed, where $$V_{t} $$ is the set of *K* vertices representing *K* topics at time *t* and $$E_{t}$$ is the set of edges at time *t*. As previously mentioned, *K* equals 80 in this paper. As shown in Eq. (3), an edge between two topics $$e_{ijt}$$ for $$1 \le i,j \le n$$ and $$i \ne j $$ at time *t* is established if their divergence $$JS(\beta _{it}||\beta _{jt})$$ in terms of topic-word distribution is below a certain predefined threshold, $$\kappa $$. In this paper, we have included a general guideline which is similar to an ‘empirical rule’ in Statistics to filter out abnormally large JS divergence values. After constructing the dynamic topic networks, we propose using the mean and standard deviation of the median JS divergence of distance pairs to define the threshold, $$\kappa $$. Our data analysis on the two cases of the 2015–2016 stock market selloff and the COVID-19 pandemic indicates that setting $$\kappa $$ at three standard deviations above the mean highlights topological properties of the dynamic topic networks which are able to generate early warning signals of severe financial market downturns or systemic risk. As in an early paper^44^, we have also conducted the sensitivity analysis to study the effect of the threshold on the topic network connectedness. Figure 11 shows the result during the 2015–2016 stock market selloff. The signals from using different thresholds are largely consistent in the sense that we see obvious drops in the average degree of the topic networks in March and May 2015, though the empirical rule of ‘three standard deviations above the mean’ may give a better result or a clear early warning signal of severe financial adjustments. The sensitivity analysis gives us extra confidence in using the dynamic topic network methodology as an additional tool for assessing systemic risk.

The data analysis suggested that the median JS divergence of distance pairs given a week of news articles had an mean of 0.73 and a standard deviation of 0.009 for the 2015–2016 time series, and those of 0.807 and 0.0056 for the 2019–2020 series. We preformed a sensitivity analysis to understand how the average degree of networks would change and suggested the empirical rule. In this paper, we present results by setting $\kappa$ to three standard deviations above the mean, i.e. 0.76 for the 2015–2016 stock market selloff and 0.82 for the COVID-19 pandemic.

3 $$\begin{aligned} e_{ijt} = {\left\{ \begin{array}{ll} 1 &{} JS(\beta _{it}||\beta _{jt}) \le \kappa , \\ 0 &{} \text {otherwise}. \end{array}\right. } \end{aligned}$$

Average degree $D_t$ as shown in Eq. (4) is a straightforward measure to calculate how many edges $|E_t|$ are in $G_{t}$ with $|V_t|$ vertices.

4 $$\begin{aligned} D_t = \frac{2|E_{t}|}{|V_{t}|} = \frac{2 |E_t|}{K}. \end{aligned}$$

To measure the degree to which the nodes tend to be linked together at time *t*, the average clustering coefficient $C_t$ is calculated. For any vertex *i*, its neighbourhood $H_{it}$ can be defined as $H_{it} = \{j: e_{ijt} \in E_t \}$. The number of vertices of $H_{it}$ is defined as $k_{it}$. Therefore, the local clustering coefficient $C_i^{local}$ for vertex *i* is calculated as

5 $$\begin{aligned} C_{it}^{local} = \frac{2|\{e_{jkt}: j, k \in H_{it}, e_{jkt} \in E_t\}|}{k_{it}(k_{it}-1)}. \end{aligned}$$

The average clustering coefficient *C* at any given time is simply the average of the local clustering coefficients of all vertices in $G_t$:

6 $$\begin{aligned} C_t = \frac{1}{K} \sum _{i=1}^{K} C_{it}^{local}. \end{aligned}$$

### Visualisation

pyLDAvis was used to help visualise the topic model in an interactive manner. Topics were projected on a two-dimensional space using principal coordinates analysis, in which the distance matrix was created by calculating the JS divergence between topic-term distributions $\beta _t$. The importance of topic *k* is represented by its node size, $A_k \propto N_k / \sum _{k} N_k$, proportional to the number of tokens generated by the topic across the corpus, with $N_k = \sum _{d} \theta _{dk} N_d$, where $N_d$ denotes the number of words in document *d* and *d* = 1, 2, ..., $M_t$. There is a relevance metric $\lambda$ to control the ranking of terms. It has a range of $0\le \lambda \le 1$, with 1 indicating terms are ranked on the basis of their topic-specific probability in a descending order and 0 meaning terms are entirely ranked by their *lift*, a term’s topic-specific probability over its marginal probability across the whole corpus. In this paper, $\lambda$ is set to 0.6 in accordance with the literature^50^.

## Data Availability

The datasets generated during and/or analysed during the current study are not publicly available due to the license of the datasets used, but are available from the corresponding author on reasonable request.
